# Design optimization of groundwater circulation well based on numerical simulation and machine learning

**DOI:** 10.1038/s41598-024-62545-7

**Published:** 2024-05-20

**Authors:** Zhang Fang, Hao Ke, Yanling Ma, Siyuan Zhao, Rui Zhou, Zhe Ma, Zhiguo Liu

**Affiliations:** Key Laboratory of Groundwater Resources and Environment, Ministry of Education, Jilin University, Changchun, 130021 People’s Republic of China

**Keywords:** Groundwater circulation well, optimization design, Numerical simulation, Machine learning, Artificial neural networks, Support vector machine, Environmental sciences, Hydrology

## Abstract

The optimal design of groundwater circulation wells (GCWs) is challenging. The key to purifying groundwater using this technique is its proficiency and productivity. However, traditional numerical simulation methods are limited by long modeling times, random optimization schemes, and optimization results that are not comprehensive. To address these issues, this study introduced an innovative approach for the optimal design of a GCW using machine learning methods. The FloPy package was used to create and implement the MODFLOW and MODPATH models. Subsequently, the formulated models were employed to calculate the characteristic indicators of the effectiveness of the GCW operation, including the radius of influence (R) and the ratio of particle recovery (Pr). A detailed collection of 3000 datasets, including measures of operational efficiency and key elements in machine learning, was meticulously compiled into documents through model execution. The optimization models were trained and evaluated using multiple linear regression (MLR), artificial neural networks (ANN), and support vector machines (SVM). The models produced by the three approaches exhibited notable correlations between anticipated outcomes and datasets. For the optimal design of circulating well parameters, machine learning methods not only improve the optimization speed, but also expand the scope of parameter optimization. Consequently, these models were applied to optimize the configuration of the GCW at a site in Xi’an. The optimal scheme for R (Q = 293.17 m^3^/d, a = 6.09 m, L = 7.28 m) and optimal scheme for Pr (Q = 300 m^3^/d, a = 3.64 m, L = 1 m) were obtained. The combination of numerical simulations and machine learning is an effective tool for optimizing and predicting the GCW remediation effect.

## Introduction

Industrial activities have intensified groundwater pollution at the global scale. Therefore, groundwater remediation has become a focus in the field of environmental sciences. Groundwater Circulation Well (GCW) represent one of the most promising techniques for in situ remediation^1,2^. The working principle of GCW is that through the structure of the well itself (mainly composed of a pumping screen section, solid section, and injection screen section), a stable three-dimensional hydraulic circulating belt is formed in the area around the well; this drives the pollutants into the pumping screen, and then the injection screen injects the clean groundwater into the aquifer to enable the removal of organic pollutants from the groundwater^3^. GCW induces a groundwater circulation zone that “sweeps” the aquifer, which may create flux across lower permeable units^4^. In addition, their remediation performance can be improved by coupling with chemical approaches, such as Soil Vapor Extraction (SVE) and bioventing^5^. Owing to these advantages, GCW has been employed globally for remediating groundwater contaminated by various pollutants. However, the optimal parameters of GCW are not well understood at defined test sites. In this study, we propose an optimization design method based on numerical simulation and machine learning to establish a GCW optimization strategy for the test site.

GCW systems have strict specifications, and to develop an effective recirculation cell, engineering decisions must be made according to site-specific criteria before selecting a GCW system^6^. Consequently, this technology still includes numerous limitations. GCW are intrinsically sensitive to hydrogeological conditions, such as horizontal conductivity (K_H_), vertical heterogeneity (K_H_/K_V_), and aquifer thickness^7^. Furthermore, the efficacy of GCW depends on operation and configuration, which are determined by parameters, such as the pumping rate (Q), length of screen sections, and separation distance between screens^8^. Therefore, the design and operation efficacy of GCW require continuous development and improvements.

The influence zone of a GCW plays a crucial role in the determination of well placement and design of a treatment system or network of GCWs. Large influence zones are considered important for site remediation. Proposed indicators to express the influence zone include the radius of influence (ROI), zone of influence (ZOI), and hydraulic capture efficiency (Pe). However, influencing factors are highly complex and further research is required to clarify the quantitative relationships between the indicators and factors at specific sites. Various studies have analyzed the practical application cases of GCW by establishing structural parameters and assessing their operational impact through tracer tests^9^. Physical experiments, field site tests, and numerical simulations are the most common measures for confirming these indicators^7,10–13^. Among them, numerical simulations are sophisticated and applicable for broad scenarios. They play important roles in estimating influence indicators in order to guide installation at real sites with complex hydrogeological conditions^8,13,14^. The Finite-Difference Method (FDM) and Finite-Element Method (FEM) are the most frequently used numerical techniques in groundwater flow simulation. Particle-tracking and node-dependent finite difference methods have also been employed for the design and remediation prediction of GCW systems in confined aquifers^11,15,16^.

Numerical simulation is used to predict GCW performance and analyze the influence of structural and hydrogeological parameters on the influence range with regards to the remediation of contaminated sites^17^. However, it is difficult to determine the quantitative relationships between the circulation effect and its influencing factors under general conditions. To ensure optimal GCW performance by enhancing the influence zone and particle recovery ratio, it is crucial to factor in specific hydrological conditions and GCW structures during their design. In general, numerical simulation is a complex, data-driven process, and parameters of the GCW are required to achieve the optimal combination, which is highly time-consuming.

Machine learning, a branch of artificial intelligence, is capable of training models with pre-existing data; the trained model can then be used to solve specific problems and extract new information from big data^18^. Furthermore, the development of convenient programming languages and mature algorithms have eased the applicability of machine learning. With the development of computer technology in recent years, machine learning has been used in groundwater research, such as water table prediction^19^, groundwater assessment and monitoring^20^, and design of contamination remediation^21–24^. Accordingly, machine learning methods have emerged as effective tools for obtaining predictive results from potential information in groundwater and environmental research. As GCW continues to be developed and site data related to GCW are accumulated, the application of machine learning to GCW technology has become possible. With machine learning methods, existing data can be used to summarize and form a functional relationship between the established index of operation efficiency and individual influencing factors. However, machine learning has not yet been used for the design optimization of GCW. Considering its strong potential, the application of machine learning can guide the optimal design of GCW for specific sites. Moreover, using this approach, the cost and time of experimentation can be reduced, and the limitations of previous research methods can be overcome to a certain degree.

In this study, we compared three machine learning models—multiple linear regression (MLR)^25^, artificial neural networks (ANN)^26^, and support vector machine (SVM)^27^—for optimizing the design of GCW parameters (Table 1). In previous GCW parameter optimization, the parameters are usually given empirically; however, in this study the machine learning method has been used for GCW optimization parameter design for the first time and very satisfactory results were obtained.

**Table 1:** Features of the three tested machine learning models.

Model	Author(s)	Advantages	Disadvantages
MLR	Korkmaz, M	Easily establishes linear causality among various variable groups, simplifying analysis	Overlooks the interaction effect and nonlinear causality
ANN	Gupta, A. K. and Guntuku, S. C	Powerful nonlinear mapping capability, allowing the approximation of any nonlinear continuous function	ANN models with missing physical models may be significantly incorrect
SVM	Esteki, S. and Naghsh-Nilchi, A. R	Establishes linear causal relationships between multiple sets of variables; can be introduced through a kernel function in the case of nonlinearity to map it to linear	Low-dimensional calculation

In this study, the FloPy package was used to create, run, and post process MODFLOW-based models. Then, characteristic indicators were calculated based on the models using Python code. The results, including influencing factors and circulating effect, were stored in a developed document that served as the dataset for machine learning. MLR, ANN, and SVM were used to train and appraise the optimization models. The trained models were then used to analyze the complex relationships between characterization indicators and influencing factors. Finally, a typical site in Xi’an (Shaanxi Province, China) was taken as an example for optimizing the structure and pumping rate of the GCW.

## Materials and methods

**Figure 1:**
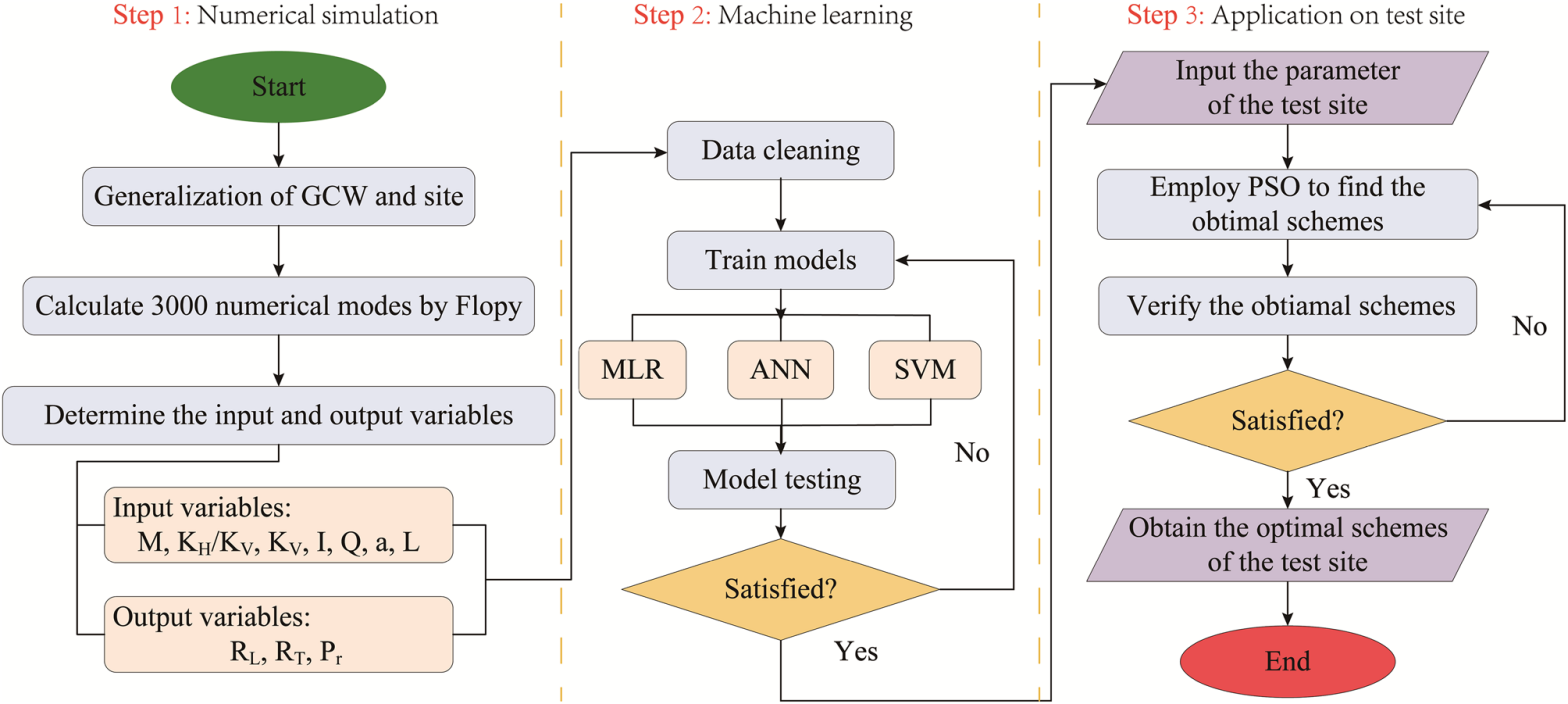
Framework of GCW optimization.

The method of GCW optimization can generally be divided into three main steps (Fig. 1). The first step is numerical simulation, in which datasets for machine learning are obtained. The second step is machine learning, in which the acquired datasets are used to train the models for optimizing the parameters of GCW. In this research, Multiple Linear Regression (MLR), Artificial Neural Networks (ANN), and Support Vector Machine (SVM) were applied to train and appraise the optimization models. In the third step, the parameters of the GCW are optimized for the test site.

### Confirmation of characterization indicators and influence factors

**Figure 2:**
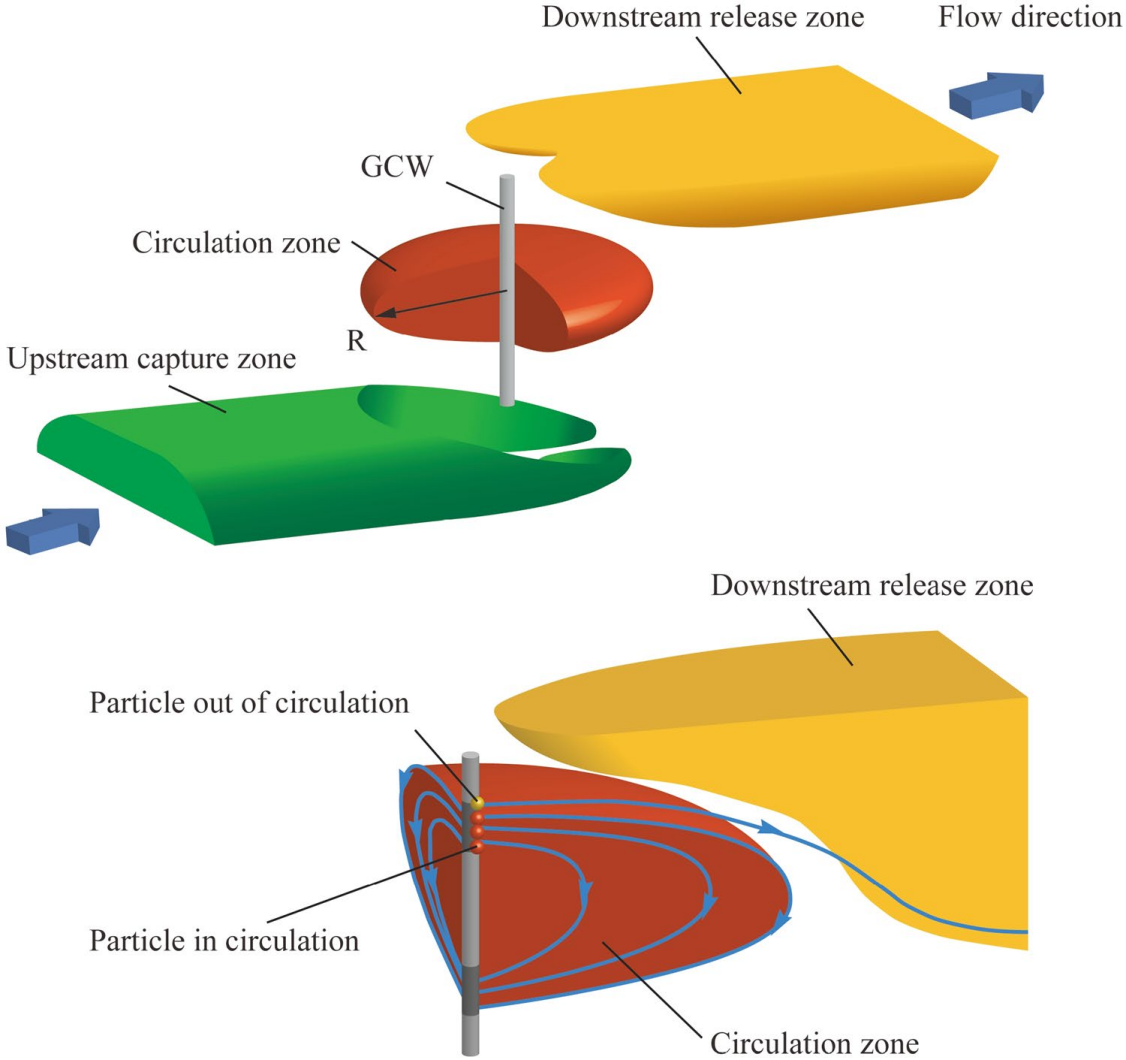
Indicators of GCW operation efficiency.

1. **Indicators of circulation efficiency:** Typically, the flow field induced by GCW exhibits the traits depicted in Fig. 2. It can be segmented into three parts, the upstream capture zone, the circulation zone, and the downstream release zone 7. In order to characterize of groundwater features surround the GCW, two indicators are usually applied^8,13,16^: the radius of influence (R) and the particle recovery ratio (P_**r**_).

**Figure 3:**
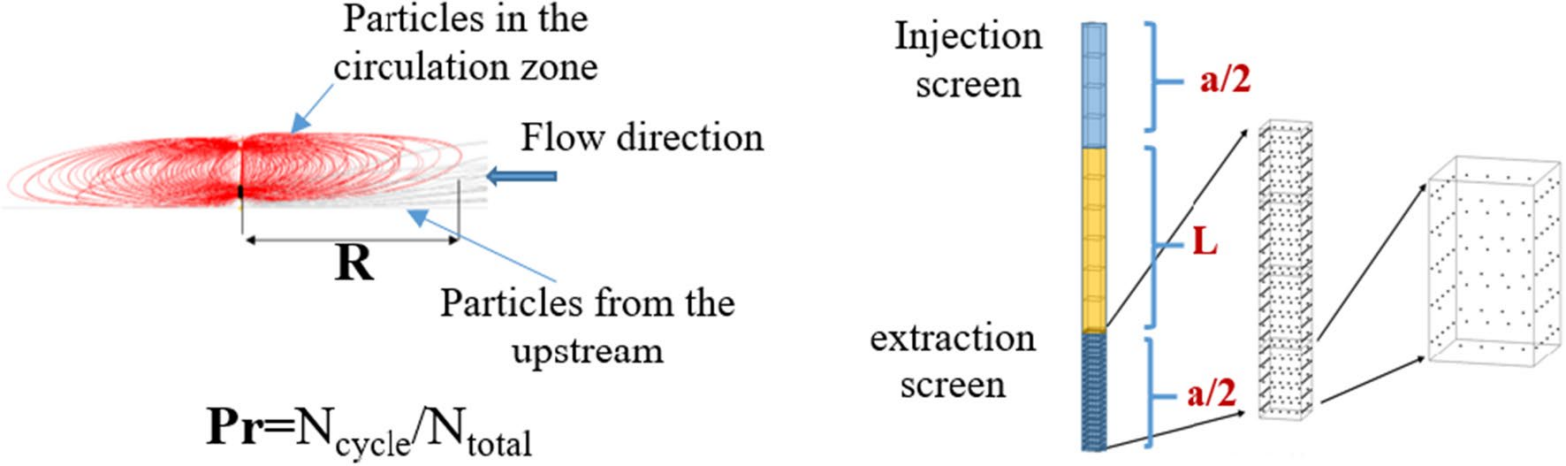
The schematic diagram of the indicators.

  ① ** Radius of influence (R):** The variable R plays a crucial role in defining the range of influence within the circulation zone. This represented the greatest horizontal separation from the circulation zone’s edge to the axis of the well. The hydraulic gradient alters the form of the circulation area, leading to fluctuating R values in different orientations. Therefore, there is a variance in the radius both along and at right angles to the groundwater flow. The indicator R, identified as the upstream radius parallel to the hydraulic gradient, is ascertainable through the computation of the distance between the particle migration trajectory in the particle tracking model.
  ② ** Particle recovery (P**_**r**_**):** The variable P_**r**_ serves as a measure for the groundwater’s capacity to be captured by the extraction screen. Groundwater from the injection screen moves towards two areas: the extraction screens and the downstream release zone. To quantify the indicator via numerical simulation, the results of particle tracking were calculated by MODPATH^12,17^. The value of P_**r**_ can be expressed as the proportion of particles in the circulation zone to the total number (N_cycle_/N_total_). The schematic diagram of the indicators is shown in Fig. 3.

2. **Influence factors:** The success of GCW operations largely stems forms the hydrogeological conditions of the remediation site. In addition, the configuration and operation mode of GCW are also crucial^8,28^. This study focused on identifying key factors influencing of GCW’s operations and their corresponding indicators of characterization. The key elements are listed below.
  ① ** Hydrogeological parameters:** Typically, hydrogeological parameters are employed to assess the appropriateness of the GCW techniques. This research primarily focused on defining hydrogeological parameters such as the horizontal conductivity (K_H_), vertical hydraulic conductivity (K_V_), the vertical heterogeneity (K_H_/K_V_), aquifer thickness (M), porosity (n), specific yield (μ), and hydraulic gradient (I).
  ② **Structure and operation parameters:** The distance between the top of the upper screen section and the bottom of the lower screen section (L) and the total length of the two screen sections (a) were used as the indicators of the circulation well structure parameters; the pumping rate (Q) was used as the main indicator of operating parameters^12,16^.

### Development of database for machine learning

The machine learning database was developed utilizing the FloPy package in conjunction with Python. A variety of tools exist for developing models, encompassing Python-based packages for plotting, manipulating arrays, optimizing, and analyzing data. In particular, FloPy was chosen for creating GCW numerical models due to its adaptability in handling MODFLOW and MODPATH packages via coding^29^. By entering varied parameter values, we derived diverse characterization indicators for GCW, leading to the creation of a database. Ultimately, the FloPy program efficiently produced over 3000 samples suitable for machine learning applications. An in-depth account of how the database was developed is provided in the supplementary material.

### Machine learning approaches

According to the views of formal researchers and the preliminary work of this study^30,31^, the key indicators R and P_**r**_ were designed as the output variables. The influence variables M, Q, I, μ, n, a, and L were set as the input variables. A reliable and effective database is important to the performance and the conclusion of machine learning. So, data cleaning before training plays a crucial role in the realm of machine learning. Box plots were used to eliminate abnormal values, and NaN (not a number), rather than numerical ones, were removed. Following the cleaning of data, the database was divided randomly into two groups; after several trials, the model results were found to be optimal for 80% and 20% of the data assigned to the training and test sets, respectively. Utilizing the training dataset, the model was trained to derive the functions linking the input and output variables. The test dataset was applied to assess the model’s forecasting capabilities.

### Database preprocessing

The application of computer technology to imitate human learning activities is a relatively new field of research^18,32^. A variety of analytical techniques are utilized within machine learning algorithms to construct related models. Each of these models is employed to deduce new tasks form the data.

Generally, machine learning algorithms can be categorized into two types based on their modeling methods: supervised and unsupervised learning. Supervised learning involves training a model to elucidate the link between feature variables and their results. Conversely, in the realm into the unexplored configurations of a specific given dataset^33^. The aim of this research was to explore how characterization indicators correlate with influence factors. It is a typical regression problem. Consequently, the method involving partially supervised learning method was applied. MLR, ANN, and SVM serve as effective techniques in addressing regression issues.

#### Model training

Python, known for its readability, interactivity, and cross-platform nature, excels in code development efficiency. Scikit-learn is a package of Python that integrates a variety of advanced machine learning algorithms and can be used to solve medium-scale supervised and unsupervised problems^34,35^. In this research, three distinct algorithms (MLR, ANN, SVM) for model training within the Scikit-learn package were adopted. The theory of the algorithms are as follows.

1. Multiple linear regression

Multiple linear regression (MLR) is the most common method for determining the linear relationship between input and output variables when handling features with limited data. The MLR method was applied to find a linear correlation between input and output variables. The mathematical form is as follows^25^:

1 $$ \hat{y} = b_{1} x_{1} + b_{2} x_{2} + \cdots + b_{n} x_{n} + c $$

where $\hat{y}$ is the regressor; $b_{i}$ (*i* = 0, 1, 2, …, n) is the coefficient of each variable, which represents the weight of the variable;$x_{i}$(*i* = 1, 2, …, n) is the input variable of the regression; *c* is the intercept term. Through continuous training, the values of *b*_*i*_ and *c* are confirmed according to the minimization of the fitting error between the forecasted and actual value. The key of MLR method is the least squares method which is widely employed to estimate the parameters by fitting a function to a set of measured data. This approach seeks to identify the best outcome when the sum of squares error (*SSE*) is minimized. *SSE* can be defined as follows:

2 $$ SSE = \sum\nolimits_{1}^{n} {r_{i}^{2} } $$

where

3 $$ r_{i} = y_{i} - f(x_{i} ,\beta_{i} ) $$

*SSE* values approaching zero indicate the closeness of estimated parameters to the actual value. If $f(x_{i} ,\beta_{i} )$ is linear, then it is a linear least square. The least squares model can be solved by employing simple calculus. However, if $f(x_{i} ,\beta_{i} )$ is nonlinear, it can be solved by an iterative numerical approach 30.

2. Artificial neural networks

**Figure 4:**
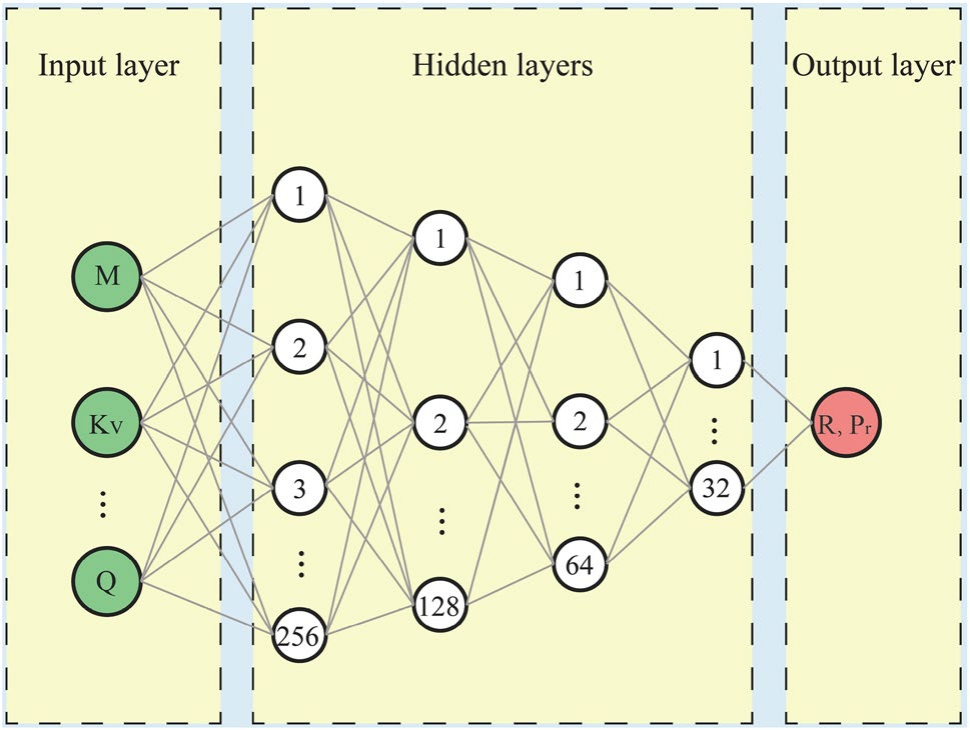
Structure of ANN for optimization of GCW.

Artificial neural networks (ANN) can execute learning and prediction functions through the emulation of human learning processes. ANN is capable of identifying links between input and output data while forming and fortifying neurons connections^26,36^. An algorithm based on a multi-layer perception neural network could rank as the top choice among artificial neural network algorithms. Due to its capability to tackle complex regression problems, this research opted to develop artificial neural networks featuring input, output, and hidden layers to enhance GCW optimization (Fig. 4).

Neurons linked in unison convert the input data into output values. In the input layer, seven neurons, symbolizing seven input variables, were established. A single neuron, symbolizing an individual goal for each predictive issue, was set in the output layer. Numerous experiments were conducted to ascertain the optimal hyper-parameters. To forecast R and Pr, four hidden layers were set, starting with 256 neurons, followed by 128 neurons in the next layer, 64 neurons in the third, and 32 neurons in the fourth. The design of the model can be described as follows:

4 $$ {\text{x}}_{t + T}^{F} = F(X_{t} ,w,\theta ,m,h) = \theta_{0} + \sum\limits_{j = 1}^{h} {w_{j}^{out} } f\left( {\sum\limits_{i = 1}^{m} {w_{ji} x_{t - i + 1} + \theta_{j} } } \right) $$

where ${\text{x}}_{t - i + 1} ,i = 1,...,m$ represents the element of the input vector $X_{t}$; ${\text{w}}_{ji}$ is the weight determining the relationship between the nodes; $\theta_{0}$ is the bias of the output node; $f( \cdot )$ is the transfer function. Following extensive experimentation, the optimal hyper-parameter was ascertained. The Rectified Linear Unit (ReLU) serves as the ideal transfer function for forecasting R (Eq. 5). Yet, in predicting P_**r**_, tanh (Eq. 6) ought to serve as the optimal transfer function. By utilizing neural networks, the value of loss progressively diminishes and reaches stability following 110 interactions. Consequently, the maximum number of iterations that yield reliable predictive outcomes for models ought to be 110.

5 $$ f(x) = \max (x,0) $$

6 $$ y = \tanh (x) = \frac{{e^{x} - e^{ - x} }}{{e^{x} + e^{ - x} }} $$

3. Support vector machines

The Support Vector Machines (SVM) employs the kernel functions to convert the data into a hyperspace, enabling the representation of intricate patterns^36–39^. With the emerging hyperspace, SVMs aim to develop a hyperplane suitable for categorizing and constructing the broadest data margin, or one that accommodates data with minimal complexity and reduced empirical risk associated with the modelling function^27^. SVMs have been applied recently for many purposes in the field of hydrogeology^40–42^. In this study, the training data can be presented as {(*x*_*i*_*, y*_*i*_), *i* = 1, 2,3, …, *n*}, where *x* is the input variable, and *y* is the output variable. A loss function offered by the SVM can be delineated in the following manner^43–45^:

7 $$ L_{\varepsilon } (y,f(x,\omega )) = \left\{ \begin{gathered} \, 0 \quad if\left| {y - (\omega \phi {(}x{) + }b)} \right| \le \varepsilon \hfill \\ \left| {y - (\omega \phi (x){ + }b)} \right| - \varepsilon \quad otherwise \hfill \\ \end{gathered} \right. $$

**Figure 5:**
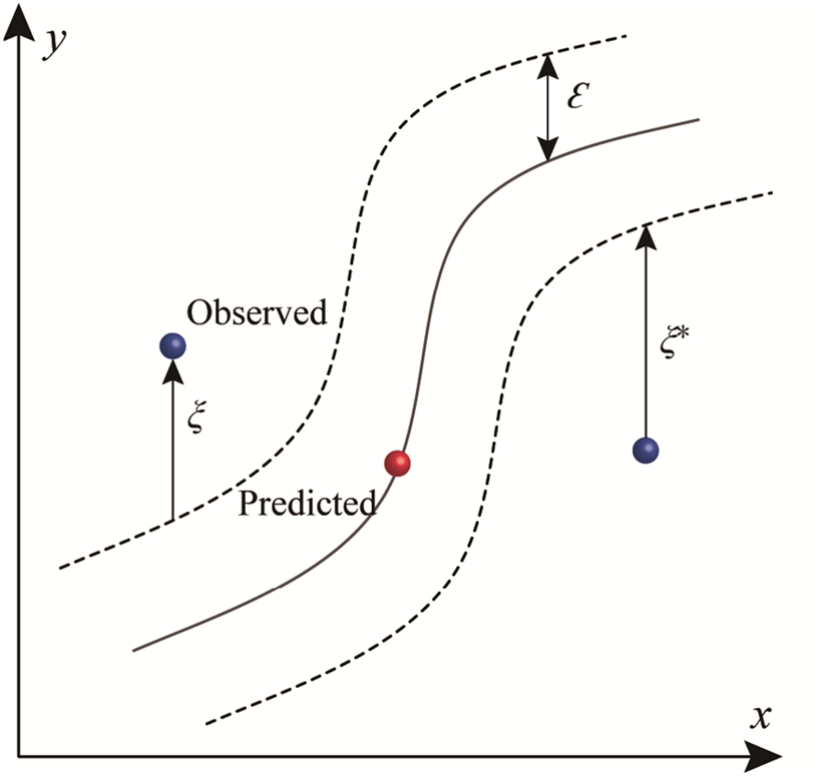
Support vector regression.

The issue with SVM can be characterized as the following optimization problem:

8 $$ {\text{minimize}}\;\;R_{{\omega ,\xi_{i}^{{}} ,\xi_{i}^{*} }} = \frac{1}{2}\left\| {\left. \omega \right\|} \right.^{2} + C\sum\limits_{i = 1}^{n} {\left( {\xi_{i}^{{}} + \xi_{i}^{*} } \right)} $$

9 $$ {\text{subject to}}\;\;\left\{ \begin{gathered} {\text{y}}_{{\text{i}}} - f(\phi (x_{i} ),\omega ) - b \le \varepsilon + \xi_{i}^{{}} \hfill \\ f(\phi (x_{i} ),\omega ) + b - y_{i} \le \varepsilon + \xi_{i}^{*} \hfill \\ \xi_{i}^{{}} ,\xi_{i}^{*} \ge 0 \hfill \\ \end{gathered} \right. $$

where $\phi \left( x \right)$ is a kernel function designed for projecting the data into a hyperspace; $\frac{1}{2}\left\| \omega \right\|^{2}$ stands for generalization; $C\mathop \sum \limits_{i = 1}^{n} \left( {\xi_{i} + \xi_{i}^{*} } \right)$ represents empirical risk; $\xi_{i}$ and $\xi_{i}^{*}$ are slack variables for measuring “below” and “above” the $\varepsilon$ tube (Fig. 5). Slack variables hold positive values while $C$ remains a positive constant.

#### Model testing and comparison

The testing dataset was used to test the MLR, SVM, and ANN models by comparing their performance with statistical measures. The precision was evaluated by computing the coefficient of determination (R^2^) and Root Mean Square Error (RMSE) using the fitting curve. A near-1 absolute value of R^2^ suggests enhanced precision within the model. When the RMSE value nears 0, there is an enhancement in the model’s fit. Their mathematical formulas are as follows:

10 $$ R^{2} = \frac{{\sum\limits_{i = 1}^{n} {(\hat{y}_{i} - \overline{y})} }}{{\sum\limits_{i = 1}^{n} {(y_{i} - \overline{y})^{2} } }} $$

11 $$ RMSE = \sqrt {\frac{1}{n}\sum\limits_{i = 1}^{n} {(\hat{y}_{i} - y_{i} )^{2} } } $$

where $\hat{y}_{i}$ is the estimated value; $y_{i}$ is the actual value; n is the number of actual values.

## Application on test site

**Figure 6:**
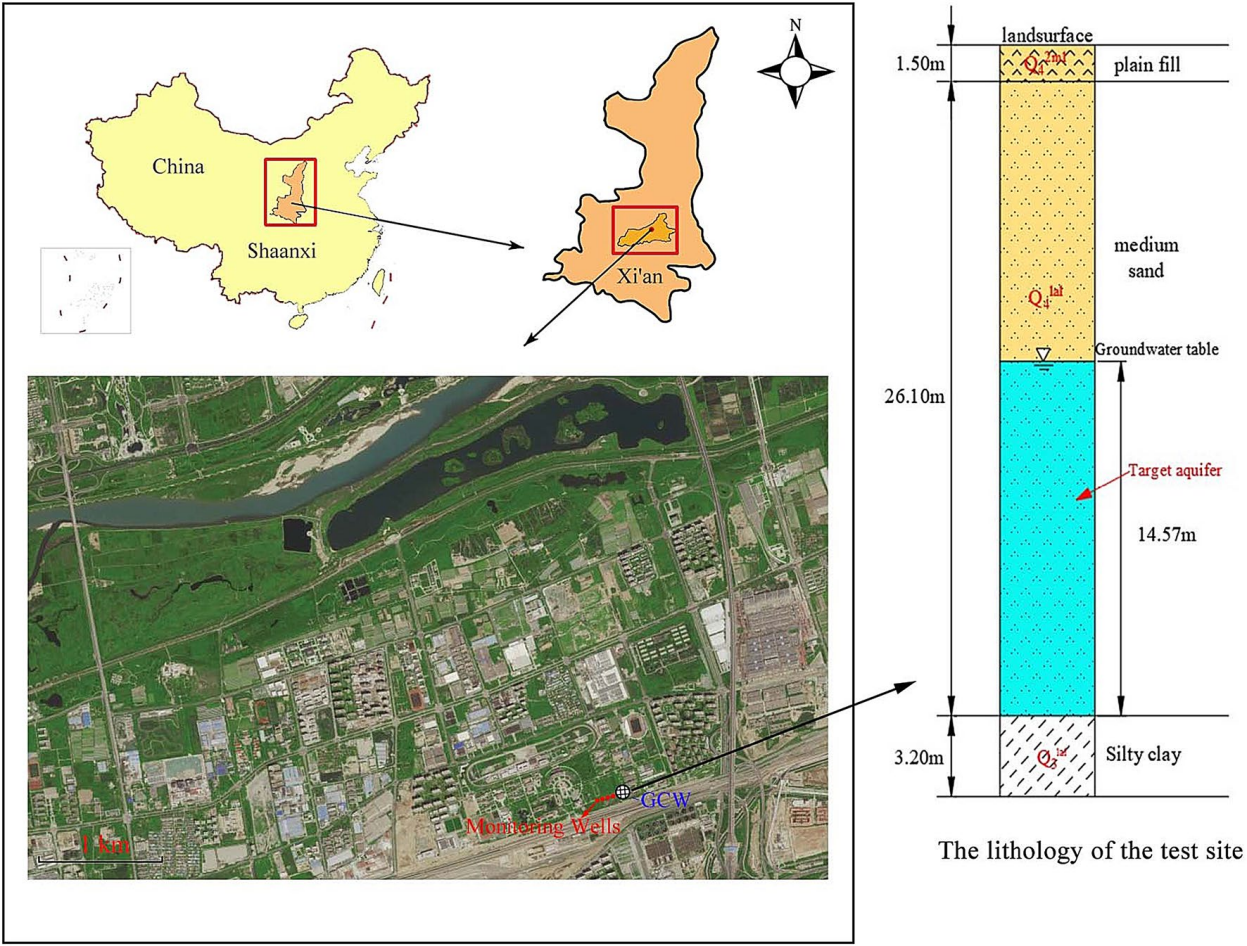
Location of the GCW and the lithology of the test site.

The test site for this study is located in Xi’an City, Shaanxi Province (Fig. 6). The area has a warm temperate continental monsoon climate, with an annual average temperature of 13.6 °C and an average annual precipitation of 732.9 mm, mainly from July to September. According to the results of hydrogeological surveys and pumping tests, the sediments at this site are mainly coarse sand, interspersed with medium and fine sand mixed with pebbles. The groundwater depth is 13.03 m. The vertical hydraulic conductivity (K_V_) is 8.33 m/d. The aquifer anisotropy of conductivity (K_H_/K_V_) is 3. Its thickness (M) stands at 14.57 m. The hydraulic gradient (I) is 0.00357. The Quaternary porous aquifer is the target aquifer for its substantial water supply capabilities. According to the information provided, the aforementioned models are suitable for enhancing the GCW parameters at the test site.

Utilizing the particle swarm optimization (PSO) algorithm, the optimal value was identified post-training models, considering its straightforwardness and superior efficacy 41, 42. It is inspired by social behavior in nature such as bird flocking. Initially, PSO consists of a multitude of particles moving through the universe, organized to identify the best solution. During each cycle, pbest and gbest modify every particle. The pbest is represents the optimal solution a particle has achieved to date, whereas gbest stands as the universally optimal solution for any particle. Once pbest and gbest have been identified, the velocity and positions of the particles can be modified in the following manner:

12 $$ V[ \cdot ] = V[ \cdot ] + c_{1} *rand( \cdot )*(pbest[ \cdot ] - present[ \cdot ]) + c_{2} *rand( \cdot )*(gbest[ \cdot ] - present[ \cdot ]) $$

13 $$ present[ \cdot ] = present[ \cdot ] + V[ \cdot ] $$

where $V\left[ \cdot \right]$ means the particle velocity; $present\left[ \cdot \right]$ represents the current particle; $rand \left( \cdot \right)$ is a random number between 0 and 1; $c_{1}$ and $c_{2}$ are defined as the learning factors, all operations of PSO algorithm are written in Python.

The aim of optimizing GCW is to identify the best solutions for models that exhibit the most rational R and Pr values. Enhancing R may expand the range of impact, whereas boosting P_r_ could improve the effectiveness of corrective measures. Aimed at optimizing the values of R and P_r_, the particle swarm algorithm was employed to identify the most suitable solution. For each defining parameter, the fitness function was derived from the design optimization model.

### Institutional review board statement

This study did not require ethical approval; all data are available in the public domain.

## Results and discussion

### Model calibration

A groundwater numerical simulation model influenced by GCW in the test site was established based on the hydrogeological survey and pumping test. The GCW was set in the center of the model, along with four observation wells been placed alongside GCW with 5 m interval in the downstream direction. The wells were used to monitor the alteration in groundwater levels around the GCW. The observation data of water level variation and numerical simulation model’s calculated data were both fitted to the water level in this study. the forward method was used to adjust the numerical model's parameters until the simulated and measured values were in close agreement. the adjusted parameters of the site are shown in Table 2.

**Table 2:** Corrected hydrogeological parameters.

Lithology	K_H_ (m/d)	K_H_/K_V_	µ	n	I
Alumina	25	3	0.2	0.39	0.00357

**Figure 7:**
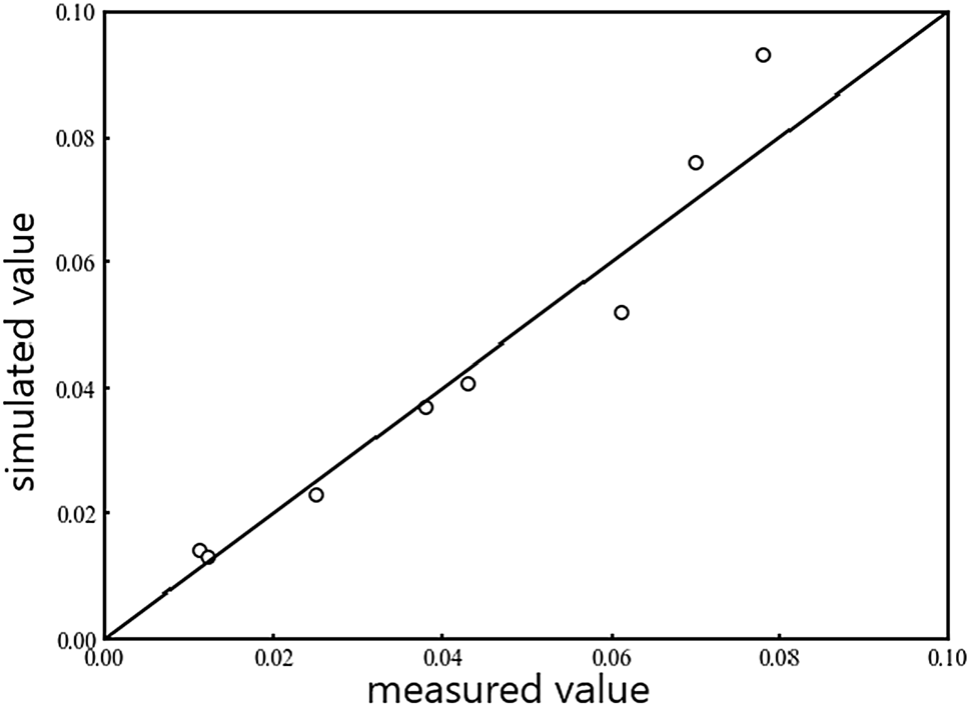
The variation of observed and the simulated water level.

The comparative outcomes of the variation of observed and the simulated water level are shown in Fig. 7. It is evident that the degree of fitting is substantial. Consequently, the proven reliability of the existing numerical simulation model is evident, suggesting that both the model and its parameters can precisely mirror the present situation, paving the way for future research.

### Database description

**Figure 8:**
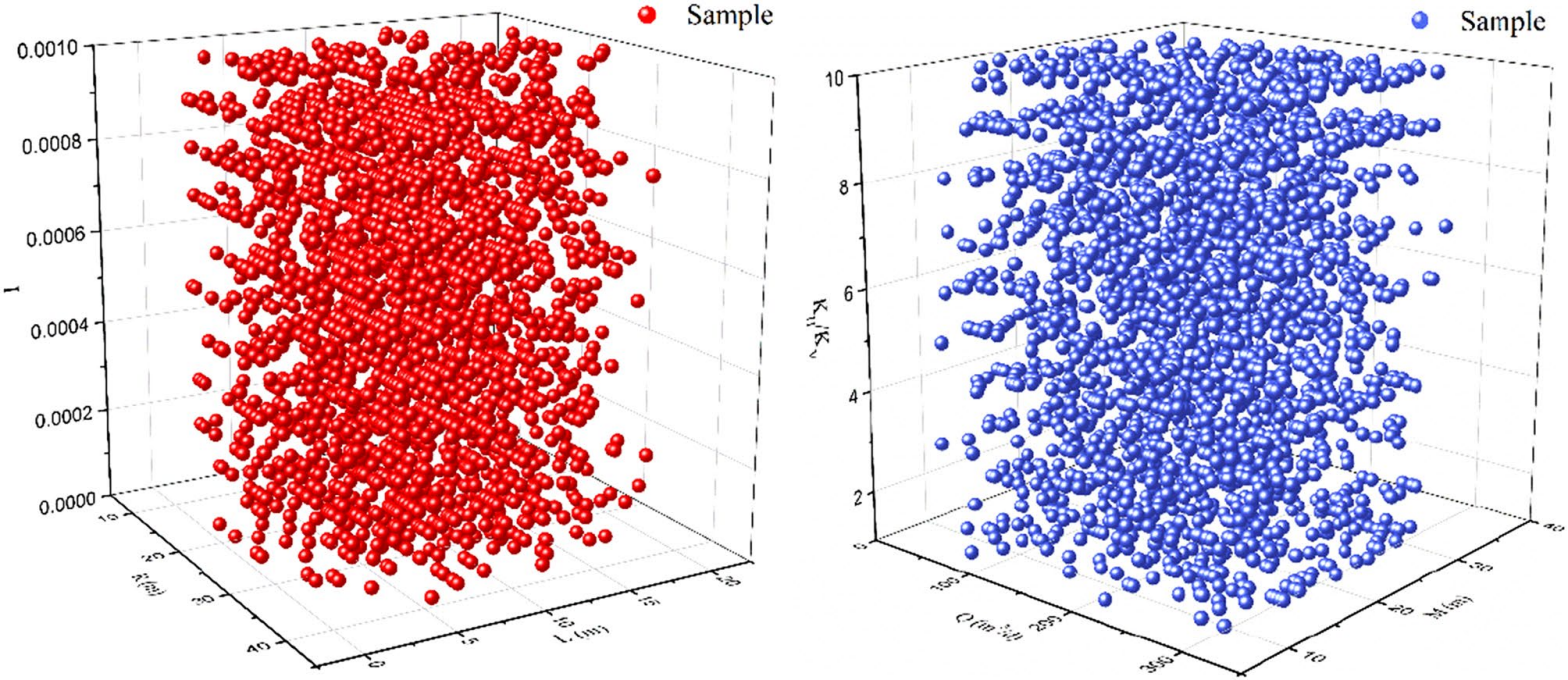
Distribution of main parameters in the database.

The database developed through numerical models in this study cover different media, aquifer thickness, and the hydrogeological parameters compositions apt for GCW 21. In these models, Q ranged from 12 to 299 m^3^/d, M ranged from 1.5 to 35 m, K_H_/K_V_ ranged from 3 to 10, K_V_ ranged from 0.5 to 34 m/d (Table 3). Figure 8 illustrates the spread of key parameters within a three-dimensional framework. The data points, symbolizing a segment of the database, are uniformly and extensively dispersed, signifying the dependability and representativeness of the database developed through numerical modeling.

**Table 3:** Allocation of key parameters along with their respective indicators within the database.

Description	Variable	Unit	Min	Max	Mean
Pumping rate	Q	m^3^/d	12	299	155.5
Aquifer thickness	M	m	1.5	35	18.25
Vertical anasitropy of hydraulic conductivity	K_H_/K_V_	–	1	10	5.57
Vertical hydraulic conductivity	K_V_	m/d	0.5	34	5.69
Total length of the two screen sections	a	m	6	24	11.07
Distance between the two screen sections	L	m	1	20	9.46
Hydraulic gradient	I	–	0	0.01	0.0005
Radius of influence	R	m	6.142	43.131	25.11
Ratio of particle recovery	P_r_	–	0.018	1	0.62

### Results of different models

**Figure 9:**
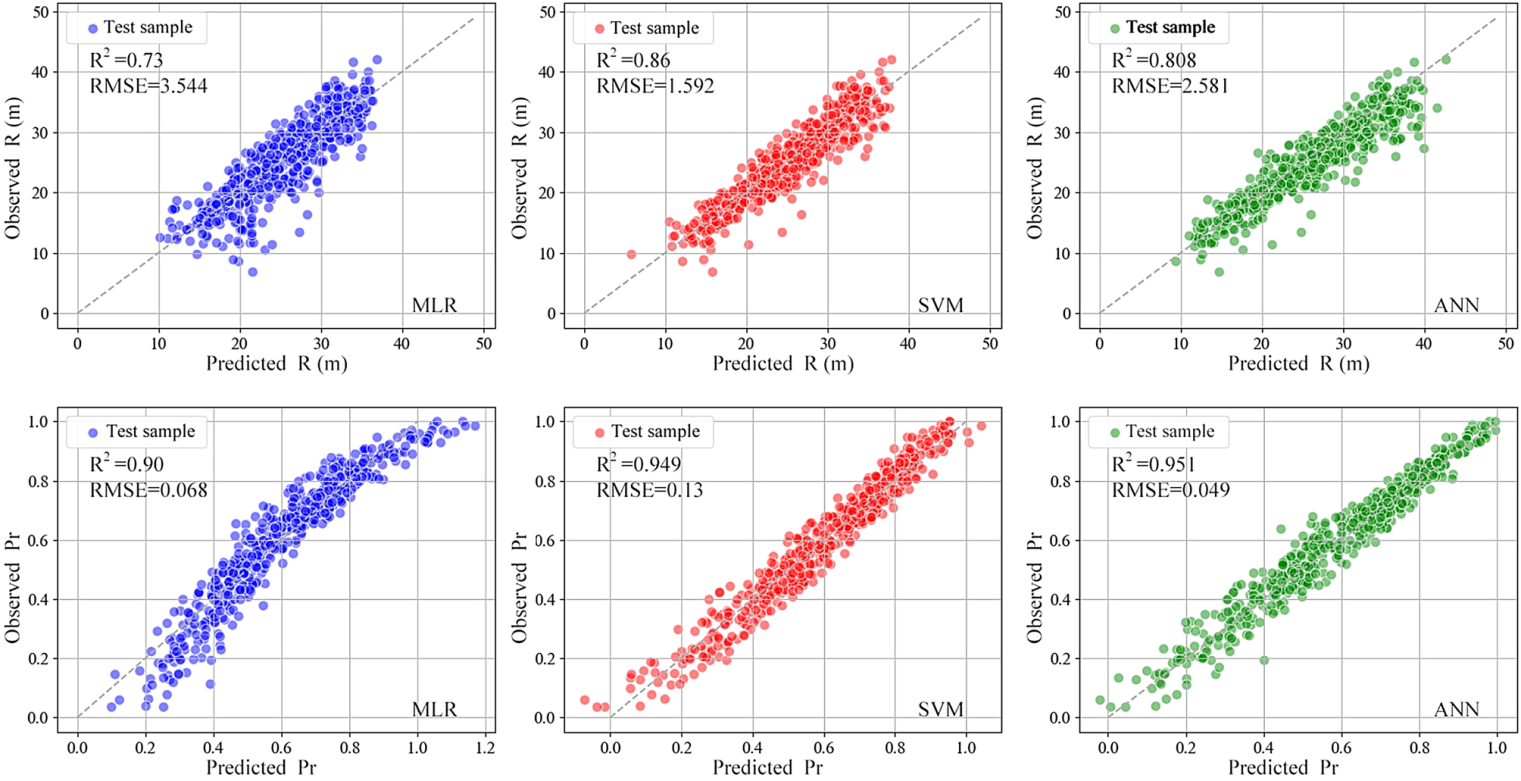
Scatter plot of Machine learning prediction.

The R^2^ and RMSE values for the SVM, ANN, and MLR models are shown in Table 4. Additionally, we entered the parameters of the testing site into the trained model to get the anticipated values of R^2^ and P_r_. Compared them with the observed data, the results are shown in Fig. 9. By comparing the prediction accuracy evaluation metrics R^2^ and RMSE, along with the scatterplots for these three models, reveals that the SVM model showed the best performance for the prediction of R^2^. Despite this, the ANN model exhibited enhanced precision in predicting Pr. As a result, the SVM and ANN models appear suitable for sequentially forecasting R and Pr throughout the design enhancement of GCW.

**Table 4:** Coefficient of determination (R^2^) and RMSE for the indicators of each model.

Forecasting indicator	R^2^			RMSE		
	SVM	ANN	MLR	SVM	ANN	MLR
R	0.86	0.808	0.73	1.592	2.581	3.544
P_r_	0.949	0.951	0.90	0.13	0.049	0.068

An analysis of the scatter points from the three distinct methods reveals a closer grouping of the SVM model's points around the y = x target line. For predicting R, the SVM model achieves the highest R^2^ value and the RMSE value nearest to 0. In predicting P_r_, the distribution of scatter points in the ANN model is denser along the target line. The ANN model has exhibits the highest R^2^ value and RMSE value nearest to 0. Therefore, the SVM model shows higher performance in the prediction of R, while the ANN model excels in predicting Pr. Compared with the previous two methods, the MLR model performs lower precision in forecasting the two indicators.

1. The MLR algorithm demonstrates greater efficacy in training scenarios with restricted datasets. Conversely, the SVM and ANN algorithms are superior in training with extensive datasets, more accurately reflecting the interaction between input and output variables^36^. Such results confirm that the optimized design of GCW is not a simple linear relationship between the parameters, but is instead a complex regression problem.
2. The simplicity of the MLR algorithm is notable, accompanied by a certain level of underfitting in its outcomes. Conversely, the SVM and ANN models provide an appropriate degree of complexity. In the context of these models, neither underfitting nor overfitting happens during the data training phase. Furthermore, their capacity for making generalize are comparatively superior^37^.
3. After comparing the three models, we found that the SVM model predicts R and the ANN model predicts P_r_ with the best results; in future studies, the two models should be integrated to predict the effects of different optimization indicators.

### Optimization of the GCW structure at the test site

**Figure 10:**
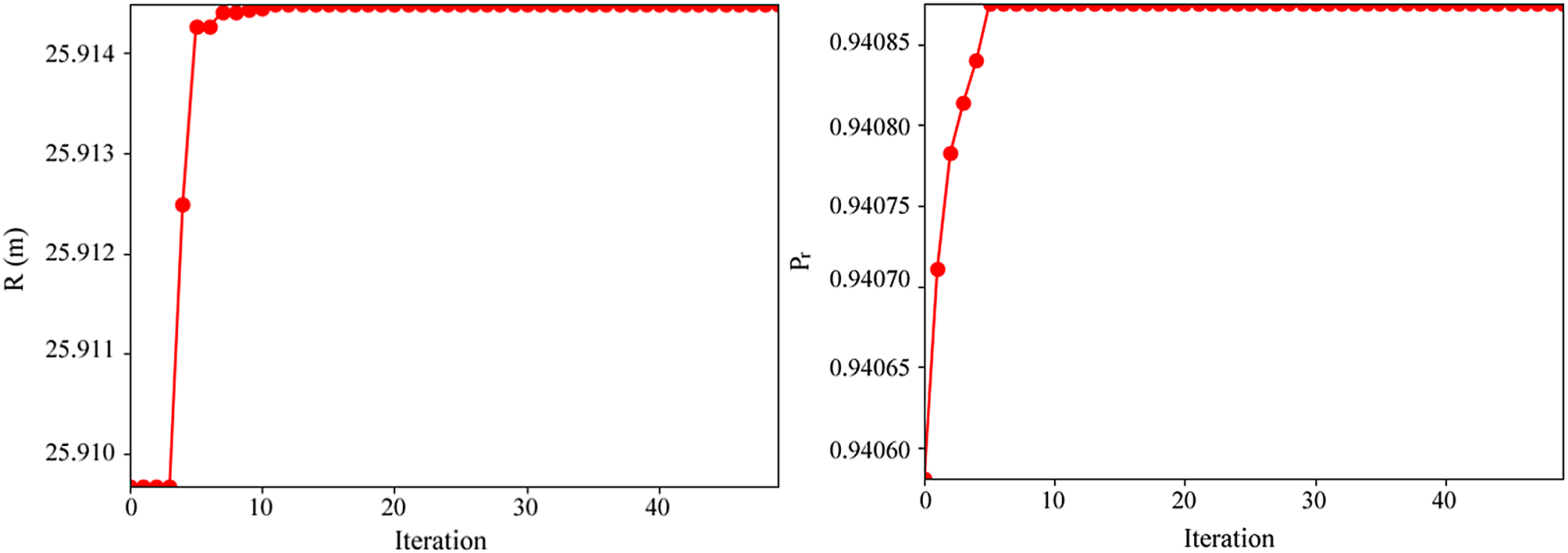
Changes in objective values with iterations.

To determine the optimal solution rapidly and accurately, a total of 1000 particles were set up. The learning factors ${c}_{1}$, ${c}_{2}$ were set to be 2.0. According to the site’s actual circumstances, specific ranges of each parameter were set. After 50 iterations, the solutions of each design optimization model were verified. The iterative processes of PSO are shown in Fig. 10. For R and P_r_, there was a fluctuation in the objective value before 10 interactions, followed by a tendency to stabilize afterwards. After 50 iterations, the target value remained almost constant. Therefore, the solution can be used as the optimization schemes after 50 iterations, evidently showing effective PSO convergence and the suitability of the chosen parameters.

The key parameters for optimization scheme are shown in Table 5. For the optimization of R, the pumping rate should be high and a relatively long distance should be maintained between the two screens in order to push particles to a far position. For the optimization of P_r_, it’s crucial to keep the pumping rate elevated and keep a minimal gap between the screens to ensure that the particles circulate in a small space with high recovery.

**Table 5:** Optimal solution of each characterization parameter.

Optimization objective	Optimal solution			Maximum value
	Q (m^3^/d)	a (m)	L (m)	
R	293.17	6.09	7.28	25.915
P_r_	300	3.64	1	0.941

Before the design optimization, several numerical models were established to set R and P_r_ with different design parameters. According to the results of the numerical simulation, a scheme with best effect was selected as an initial scheme for each indicator (Q = 200 m^3^/d, a = 6 m, L = 3 m, R = 17.85 m, P_r_ = 0.86). Comparing the initial and optimized scheme, R increased from 17.85 to 25.915, accounting for an improvement of 45%, P_r_ increased from 0.86 to 0.941, accounting for an improvement of 9.4%. Both R and P_r_ increased after optimization, reflecting the effectiveness of the design optimization based on machine learning and numerical simulation.

This study proposes two optimization schemes for R and P_r_ respectively. The schemes provide designers with diverse options. In cases where the test location exhibits a broad spectrum of contamination, the optimization scheme for R is suggested to be selected for enhancing the remediation range of the GCW. When the spread of contaminants is limited and the remediation duration is urgent, the optimal scheme for P_r_ should be chosen to enhance the remediation efficiency.

## Conclusions

This paper presents a novel design approach for optimization of GCW by combining the numerical simulation and machine learning. Numerical simulation is proved to be a superior and more economical method for data collection compared to typically lengthy and expensive lab and field tests. This method enables the rapid collection of comprehensive datasets for machine learning applications. The dataset is developed based on the prevailing conventional hydrogeological circumstances. In order to expand the implementation of the proposed schemes to additional test sites in the future, the simulation of data can be conducted by considering specific hydrogeological conditions of the respective remediation sites. By exerting these efforts, machine learning models can be made more dependable and precise. This finding of this research is as follows:

1. With the consideration of the unpredictability of hydrogeological parameters, configuration of the well, and operating parameters, the MLR, ANN, and SVM-based predictive model in the machine learning algorithm exhibits excellent compatibility with the numerical simulation model of GCW. The input–output relationship of the groundwater simulation model can be accurately represented. By designing GCW optimally, we can develop machine learning models to lessen computational demands.
2. The operation efficacy of GCW plays a crucial role in groundwater remediation. In this study, two optimal strategies were implemented for GCW optimization at a test site in Xi’an. The schemes are derived from numerical simulation and machine learning using data of the test site. In order to apply schemes to other sites, only the hydrogeological parameters (M, K_V_, K_H_/K_V_, I) and the range of design parameters (Q, a, L) of the site need to be determined. By employing the trained model, we can perform calculations to improve the design of the GCW structure for specific locations. This approach provides a practical and highly efficient method for optimizing the design of GCW.

As a remediation technology of contaminated groundwater, the operational effectiveness of GCW is affected by the water quality and sediment concentration. However, the operation of GCW continues to face significant challenges due to hydrodynamic factors. During pumping and injection process, there is an increase in the hydraulic gradient of the groundwater flow field, propelling the movement of pollutant. Furthermore, it is essential to cleanse the circulation well while constructing it and use filter media around the edges of the pumping and injection screens to ensure the well’s sediment levels remain low. To sum up, the study concentrated on hydraulic circulation’s impact on GCW functioning, disregarding the influence of water quality and sediment density.

In spite of the simplified conceptual model composed of homogeneous and geometrically regular aquifer, this study is of great importance due to its considerably new scientific and practical application. In the future, more intricate situations may be considered, such as the direction of water flow. As the complexity of aquifer conditions increases, it may significantly increase the nonlinearity of the input–output relationship of simulation model. In order to improve applicability of designed GCW in complex site, deep-learning method and multiple-objective optimization model will be reached in future.

### Supplementary Information

Supplementary Information.

## Data Availability

The datasets used during the current study available from the corresponding author on reasonable request.
