# Supplementary material for: Design optimization of groundwater circulation well based on numerical simulation and machine learning
Source: Sci Rep. 2024 May 20;14:11506. doi: 10.1038/s41598-024-62545-7 (PMC11106317; doi:10.1038/s41598-024-62545-7)
Supplement: Supplementary file 1 — Supplementary Information. [file 41598_2024_62545_MOESM1_ESM.doc]

# **Supplementary Material**

Basic steps for development data set induced by GCW with FloPy and Python is shown in Fig. S1.


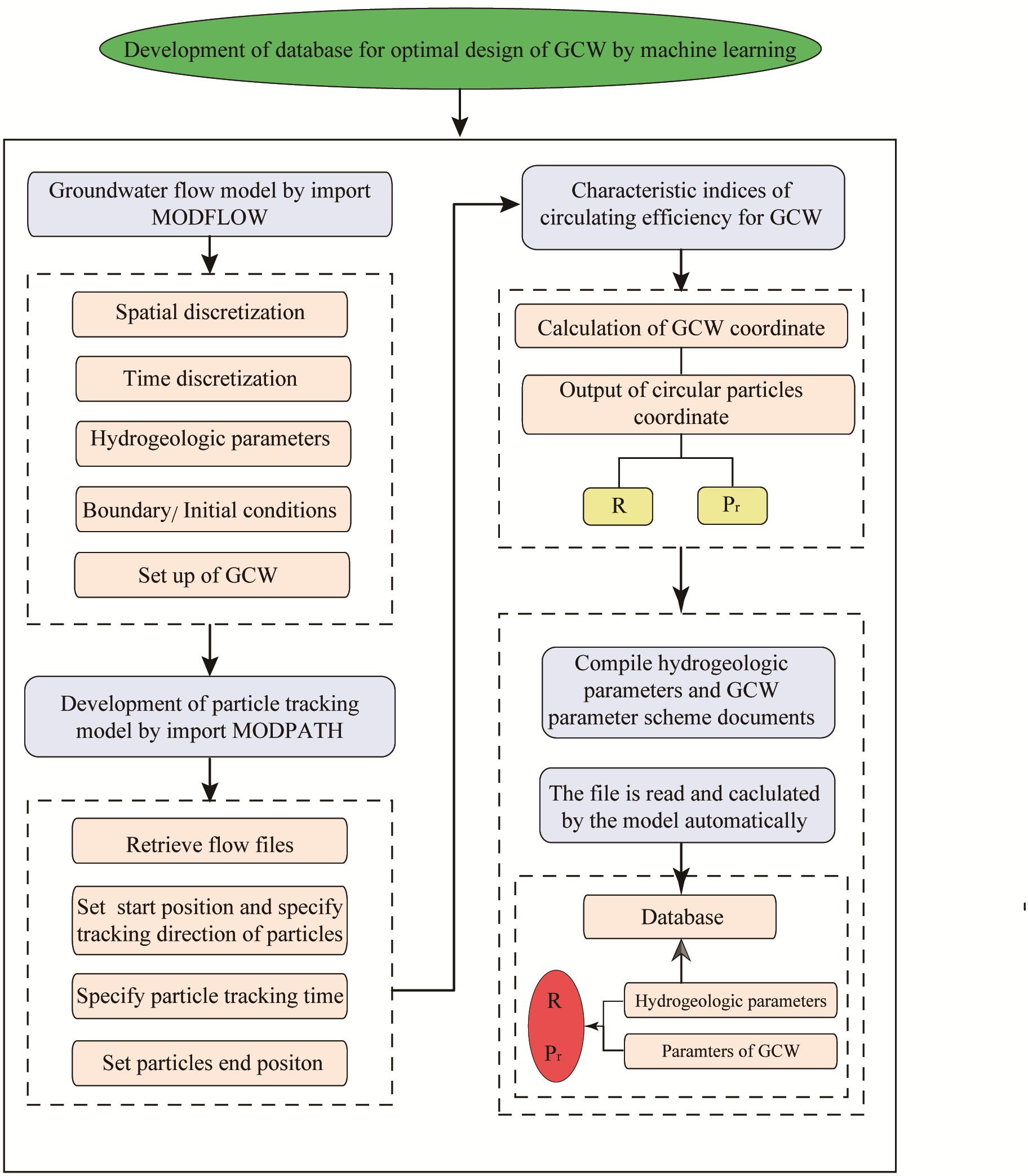


Fig. S1. Flow chart for the development of dataset corresponding to GCW

**1. Development of groundwater flow model (MODFLOW)**

(1) Spatial discretization: The initial coordinates (*X*_0_, *Y*_0_) of the simulation area in the lower left corner is entered and the scope of the simulation area is determined through the following parameters, which are the lengths *Lx* and *Ly* in the *X* and *Y* radial directions respectively, so does the top and bottom elevations (*ztop* and *zbot*) of the aquifer. Finally, the entire spatial extent is divided into a spatial grid with *nlay* layers, *nrow* rows, and *ncol* columns. The code and the result are shown in Fig. S2.


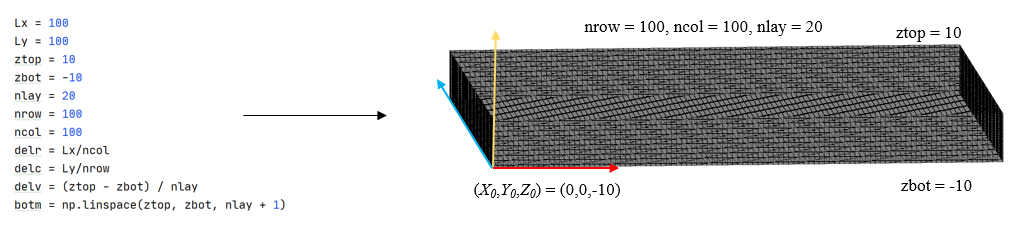


Fig. S2. Spatial discretization

(2) Time discretization: The entire simulation period is divided into *nper* stress periods. The length of each stress period is expressed as *perlen*. Each stress period is divided into several time setp which is expressed as *nstp*. The code and the result are shown in Fig. S3.


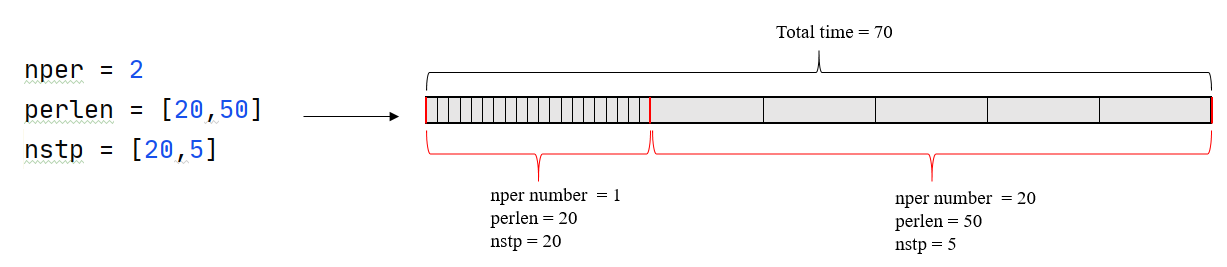


Fig. S3. Time discretization

(3) Hydrogeologic parameters: Hydrogeological parameters mainly include horizontal hydrogeologic conductivity (*K_H_*), vertical hydrogeologic conductivity (*K_V_*), the anisotropy (*K_H_/ K_V_*), specific yield (*Sy*), specific storage (*Ss*), porosity (*n*), *layer type*, flow state (*steady*/*transient*) etc. The input of hydrogeologic parameters of the aquifer is flexible and convenient in the model. It can be used to complex heterogeneous aquifers. the input of hydrogeologic parameters in heterogeneous aquifer can be expressed in the code as follow. The code and the result are shown in Fig. S4.


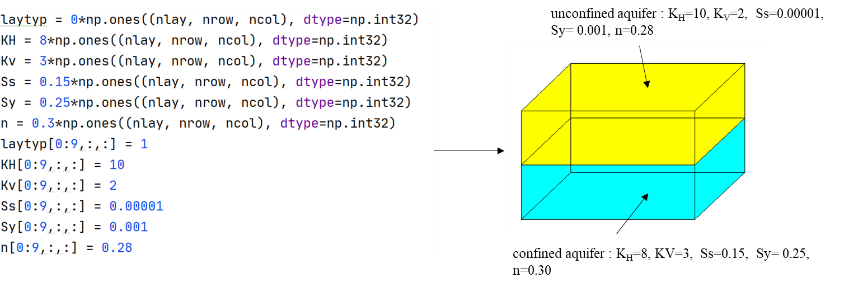


Fig. S4. Generalization of hydrogeological parameters

（4）Definite conditions. The boundary conditions and initial conditions are assigned in the form of three-dimensional arrays (*ibound*) *and* (*strt*). The corresponding cells of specific head boundary can be assigned as “-1”, the outermost impermeable boundary cells can be assigned as “-1”, and “0” refers to the inactive cells that does not need to be calculated. The code and the result are shown in Fig. S5.


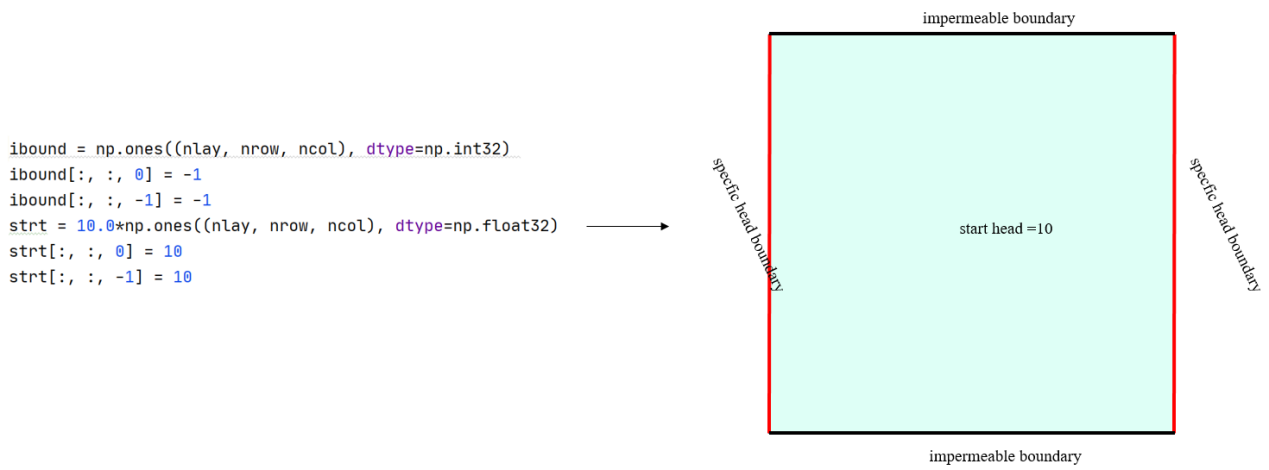


Fig. S5. Boundary conditions and initial head generalization

（5）Set up of GCW: The planed position of the GCW is specified according to the index value of the row, and the columns are located in the same depth vertically. The position of its screens is determined by four parameters: the top and bottom elevations of the upper screen, and the top and bottom elevations of the lower screen respectively. The code and the result are shown in Fig. S6.


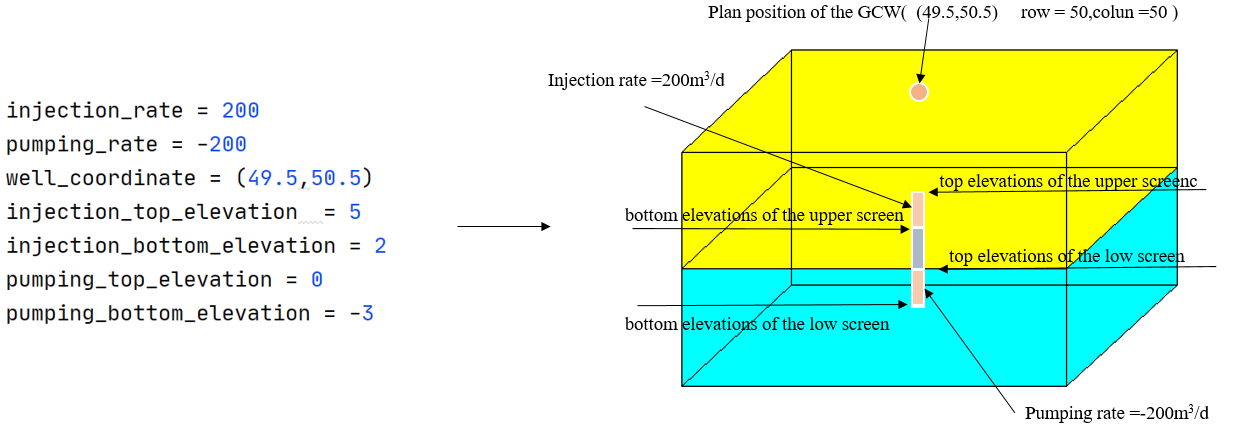


Fig. S6. Generalization of GCW parameters

After the mentioned parameters were input to the model, they are imported to Modflow packages such as ModflowDis, ModflowBas, ModflowLpf, etc. After running the MODDFOLW, the correspond files are output for particle tracking simulation in the next step. The code is shown in Fig. S7.


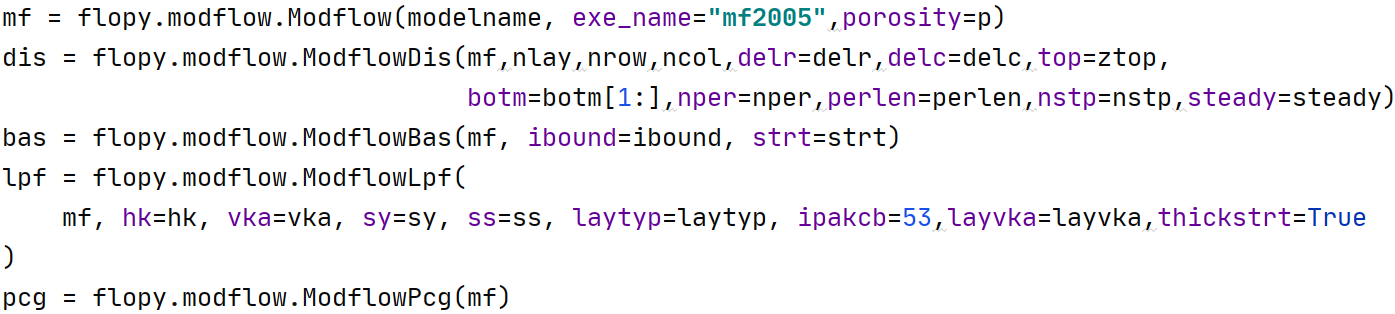


Fig. S7. Code for parameters transfer in groundwater flow model

**2. Development of particle tracking model (MODPATH)**

（1）The ModpathBas package is loaded and the Modflow parameters are imported to the model, so does the flow files induced by GCW such as spatio-temporal discrete data files, head data files, etc.


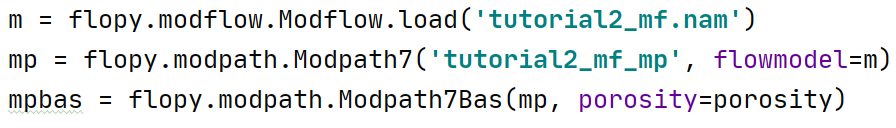


Fig. S8. Code for loading the MODFLOW data file to the ModpathBas

（2）Particles are placed at the cell faces(*Face*1*,Face*2*,Face*3*,Face*4*,*) of the injection screen and forward simulation tracking (*tracking_direction*) is selected.


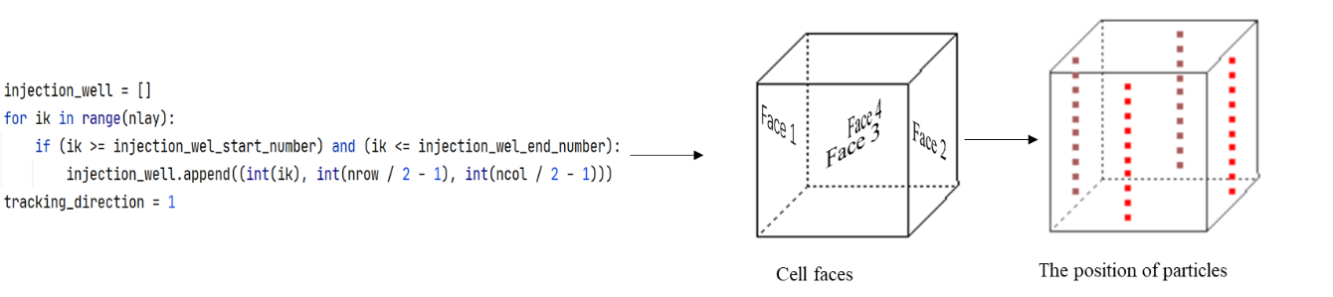


Fig. S9 particles setting

（3）The termination time for particles tracing (*stoptime*) is specified. It is set to be included in the simulation duration. The cells of extraction screens are located are assigned as the value of “2” so as to stop the movement of the particles when the particles move to the unit body code-named 2.


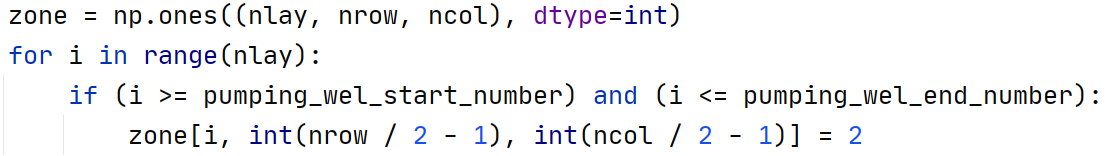


Fig. S10 Code that specifies when and where the particles end

Then the parameters mentioned above are imported to Modpath7, Modpath7Bas, ParticleData, ParticleGroup, and ModpathSim packages respectively for calculation. The corresponding particle trajectory file is exported to describe the indices for the circulating efficiency of GCW.

**3. Description of GCW for its circulating efficiency based on numerical simulation**

（1）The location of GCW is set in the center of simulation area with the coordinates (*X*_1_*, Y*_1_) (Fig. S11).

（2）In the file generated by the running of the Modpath program, the particles that terminate in the pumping screen (cells code are 2) are used as circulating particles, and the id of such particles and the coordinates (*X*, *Y*) where each time is located are output.

（3）The particles on the two faces in the *X* direction of the cell at each time node are composed into a list of data by applying Python. All the values in this list are conducted to make a difference with the radial coordinate *X_1_* of the center of GCW. Then the maximum difference value obtained is supposed to be the transverse influence radius R_T_. Similarly, the particles on the two faces in the *Y* direction of the cell at each time node are composed into a list of data by applying Python. All the values in this list are conducted to make a difference with the values in this list are conducted to make a difference with the radial coordinate *Y_1_* of the center of GCW. Then the maximum difference value obtained is supposed to be the transverse influence radius R.


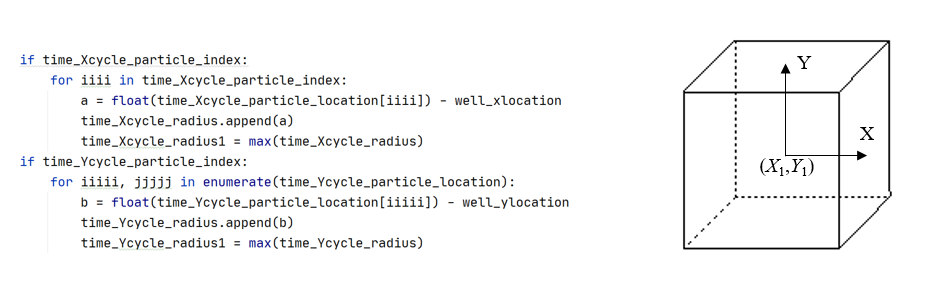


Fig. S11 Code for parameters R_T_, R_L_ characterization respectively

（4）The total number of particles set on the injection screen initially is *N*. The number of particles that migrate to the pumping screen (with the cell code “2”) within the appointed time is n. The ratio of n to N is supposed to be the Particle recovery rate (Pr*)*.


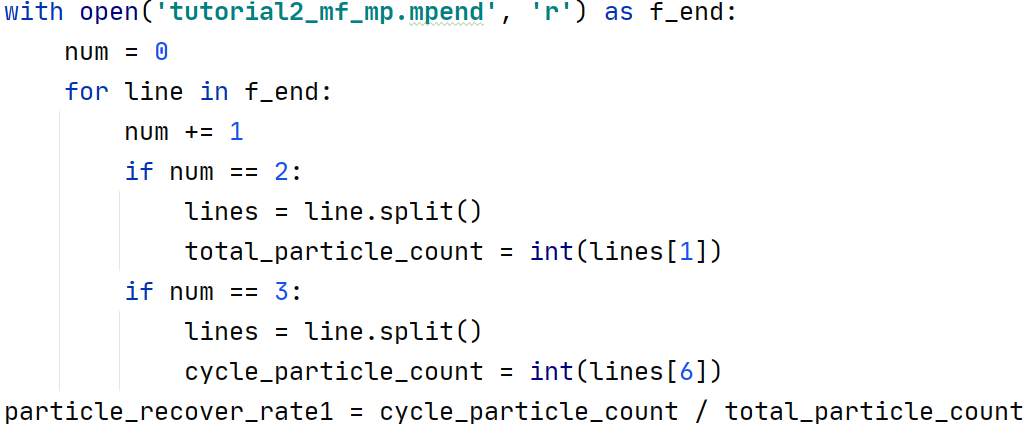


Fig. S12 Code for parameter Pr characterization

**4. Development of database**

Firstly, the *random* function is applied to create a file of combinations including hydrogeological parameters and GCW parameters. The randomness and rationality of each set of parameters are considered in this step. Then all the parameters are imported to the model separately with different combination. Numbers of groundwater numerical models were developed to get the data of R, P_r_. The data set are saved to meet the requirements of machine learning for data characteristics. The *while* function is available in the model to automatically read each set of data in the file. The indicators of circulating efficiency (R*,* P_r_) and the correspond parameters are saved in the database file in turn.

1. **Database description**

The database developed through numerical models in this study cover different media, aquifer thicknesses, and compositions of hydrologic parameters suitable for GCW. In these models, Q ranged from 12 to 299 m^3^/d, M ranged from 1.5 to 35 m, K_H_/K_V_ ranged from 1 to 10, K_V_ ranged from 0.5 to 34 m/d. The distribution of the main parameters is shown in three-dimensional space in Fig. S13.


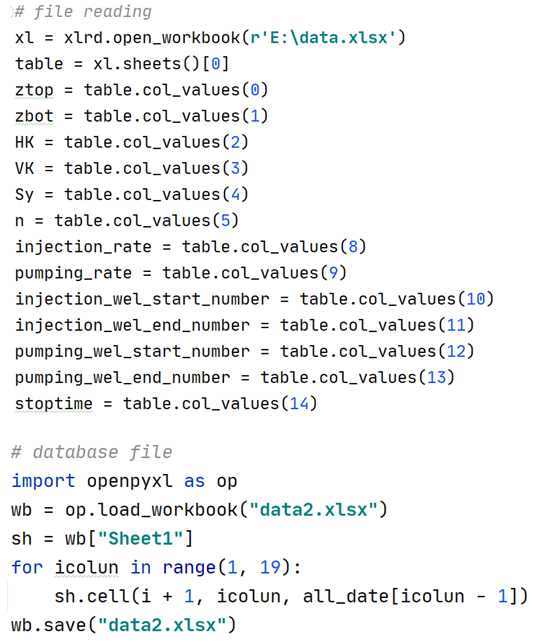


Figure S13 Code for process of the development of database
